# Resting-state fMRI graph theory analysis for predicting selective serotonin reuptake inhibitors treatment response in adolescent major depressive disorder

**DOI:** 10.3389/fpsyt.2025.1675719

**Published:** 2025-10-01

**Authors:** Xue Mo, Xuemei Li, Mengqi Liu, Linlin Hu, Qian Li, Jie Wang, Haiqing Deng, Fajin Lv, Xinyu Zhou, Yun Mao, Yang Huang

**Affiliations:** ^1^ Department of Radiology, The First Affiliated Hospital of Chongqing Medical University, Chongqing, China; ^2^ Department of Psychiatry, The First Affiliated Hospital of Chongqing Medical University, Chongqing, China

**Keywords:** major depressive disorder, adolescents, selective serotonin reuptake inhibitors, functional brain networks, graph theory

## Abstract

**Background:**

Substantial interindividual variability exists in the response of adolescents with major depressive disorder (MDD) to selective serotonin reuptake inhibitors (SSRIs), and reliable early predictors of treatment response are lacking.

**Methods:**

Resting-state functional magnetic resonance imaging (fMRI) data and clinical scale scores were collected from 69 adolescents with first-episode, drug-naïve MDD. Based on treatment response assessed after 8 weeks of SSRIs therapy, participants were categorized into a responder group (n=37) and a non-responder group (n=32). Graph-theoretical analysis was then performed on the pre-treatment resting-state functional networks of both groups.

**Results:**

Significant group differences emerged in several global attribute metrics and multiple brain region node attribute metrics (including the left middle frontal gyrus, hippocampus, parahippocampal gyrus, amygdala, pallidum, as well as the right anterior cingulate cortex and inferior parietal lobule). Partial correlation analyses revealed ​negative correlations between nodal efficiency in the left middle frontal gyrus, hippocampus, and parahippocampal gyrus, as well as degree centrality in the right anterior cingulate gyrus, and the reduction rate in Hamilton Depression Rating Scale-17 score. Furthermore, logistic regression analysis identified lower nodal efficiency in the right inferior parietal lobule and higher clustering coefficient in the left pallidum as significant predictors of SSRIs treatment response.

**Conclusions:**

Pre-treatment functional network topological metrics differentiating responders and non-responders demonstrate potential as predictors for SSRIs treatment response in adolescents with MDD.

## 1 Introduction

Major depressive disorder (MDD) is a common psychiatric disorder that severely impairs psychosocial functioning and reduces quality of life in affected individuals (1). Typically emerging during adolescence, MDD is characterized by core symptoms including persistent depressed mood, loss of interest or pleasure (anhedonia), and recurrent suicidal ideation (2). Epidemiologic studies indicate that approximately 20% of children and adolescents globally experience depressive symptoms or meet diagnostic criteria for depression, with prevalence rates exhibiting a concerning upward trend (3). Selective serotonin reuptake inhibitors (SSRIs) are currently recommended as the first-line pharmacological intervention for adolescents with MDD (4, 5). However, SSRIs exhibit a delayed therapeutic onset, typically requiring 2 to 6 weeks to become clinically apparent (6). Moreover, findings from multiple clinical studies demonstrate that the overall treatment response rate to SSRIs in adolescent MDD patients ranges only from 55% to 60% (7–9). Critically, reliable predictive biomarkers for early treatment response remain elusive.

Current longitudinal neuroimaging studies have not only confirmed that SSRIs induce structural and functional alterations in specific brain regions of MDD patients but also revealed differential changes between treatment responders and non-responders associated with symptomatic improvement (9, 10). These studies further suggest that baseline brain structure and function may serve as potential predictors of treatment outcome. Previous research utilizing multimodal MRI and clinical data with machine learning algorithms achieved a prediction accuracy of 63% for sertraline treatment response in adults with MDD (11). Similarly, another study successfully predicted the efficacy of SSRIs at 2 weeks post-treatment in adolescent MDD patients using baseline radiomic features extracted from structural MRI within a machine learning framework, yielding an AUC of 0.954 for treatment response prediction (12). However, significant limitations persist in studies predicting SSRIs response specifically in drug-naïve, first-episode adolescent MDD patients. For instance, some investigations focus solely on short-term efficacy assessment at 2 weeks (12), a time point when treatment outcomes remain unstable. Additionally, most studies concentrate on imaging analyses of single brain regions rather than whole-brain networks.

Mounting evidence highlights the role of whole-brain networks in the pathophysiology of MDD with antidepressant treatment effects distributed across multiple functional brain networks (13–15). Graph theory provides a robust framework for quantifying complex topological properties within structural and functional brain networks (16). Cross-sectional studies consistently reveal significant topological abnormalities in brain networks of adolescents with MDD (17–19), while longitudinal research further demonstrates that these networks undergo topological changes following 8 weeks of SSRIs treatment (20). Critically, graph-theoretical analyses in adult MDD populations indicate that reduced degree centrality in the dorsomedial prefrontal cortex (dmPFC) post-SSRIs treatment significantly correlates with clinical improvement (21). This evidence suggests that graph theory can identify therapy-relevant topological features with potential predictive utility for treatment response. Nevertheless, research specifically characterizing brain network topology underlying differential SSRIs responses in drug-naïve, first-episode adolescent MDD patients remains scarce.

Therefore, this study aims to employ whole-brain resting-state functional magnetic resonance imaging (fMRI) data from drug-naïve, first-episode adolescent MDD patients and apply graph-theoretic analysis to identify brain network topological features predictive of SSRIs treatment response. These findings may inform personalized treatment strategies to enhance clinical symptom management in this population. We hypothesize that: (1) Pre-treatment brain network topology significantly differs between treatment responders and non-responders following 8 weeks of SSRIs therapy; (2) Specific topological metrics correlate with clinical symptom improvement and demonstrate predictive potential for SSRIs response.

## 2 Materials and methods

### 2.1 Participants

Sixty-nine adolescent MDD were recruited through the Department of Psychiatry at The First Affiliated Hospital of Chongqing Medical University. The conduct of this study was approved by the Ethics Committee of the First Affiliated Hospital of Chongqing Medical University (Ethical approval No. 2020–864). All participants received a diagnosis of MDD based on the *Diagnostic and Statistical Manual of Mental Disorders*, Fifth Edition (DSM-5), confirmed through Structured Clinical Interviews (SCID) conducted by two board-certified psychiatrists. Demographic and clinical information was systematically collected for all included patients. Participants met the following criteria: (1) aged 13–18 years; (2) The 17-item Hamilton Depression Rating Scale (HAMD-17) score >7; (3) first depressive episode and psychotropic medication-naïve; (4) right-handed; (5) absence of severe medical/neurological conditions, psychiatric disorders other than MDD, substance abuse/dependence, or head trauma with loss of consciousness; and (6) no comorbid psychotic disorders—with the exception that anxiety comorbidity was permitted if MDD was the principal diagnosis and primary reason for clinical presentation; (7) Individuals with MRI contraindications were excluded. Written informed consent was obtained from all adolescent participants and their legal guardians.

### 2.2 Symptom assessment and grouping

HAMD-17 and Hamilton Anxiety Scale (HAMA) were administered to evaluate Severity of the patient’s depressive and anxiety symptoms. All patients underwent 8 weeks of SSRIs treatment and were subsequently stratified into responder (n=37) and non-responder (n=32) groups based on HAMD-17 score reduction rates (≥50% for responders; <50% for non-responders). The HAMA score is primarily used to describe pre-treatment clinical characteristics of the sample and to assess comparability between responder and non-responder groups regarding baseline anxiety levels in subsequent analyses.

### 2.3 MRI data acquisition

All participants underwent scanning using a 3.0-T MRI system (Skyra, Siemens Healthcare, Erlangen, Germany) with a 32-channel head coil, where foam pads and earplugs were utilized to minimize head motion and attenuate scanner noise. Participants were instructed to remain relaxed with closed eyes while maintaining wakefulness; no subjects reported discomfort or sleep onset during scanning. Conventional axial T2-weighted and fluid-attenuated inversion recovery (FLAIR) images (5-mm slice thickness) were acquired for lesion screening, followed by whole-brain resting-state fMRI data acquisition via gradient-echo echo-planar imaging (GRE-EPI) sequence with these parameters: 36 axial slices; 3-mm slice thickness (no gap); repetition time (TR)=2,000 ms; echo time (TE)=30 ms; flip angle=90°; matrix=64 × 64; voxel size=3.4 × 3.4 × 3 mm³; field of view (FOV)=220 × 220 mm². This 8-minute fMRI scan yielded 240 volumes per participant. High-resolution structural images were then obtained using a magnetization-prepared rapid gradient-echo (MPRAGE) T1-weighted sequence with parameters: 192 sagittal slices; 1-mm slice thickness (no gap); TR=2,000 ms; TE=2.56 ms; flip angle=9°; matrix=256 × 256; isotropic voxel size=1 × 1 × 1 mm³; FOV=256 × 256 mm². Finally, two radiologists performed visual quality control on all images to exclude lesions and artifacts.

### 2.4 Data processing

DICOM raw images were converted to NIFTI format using dcm2nii software. Resting-state fMRI data preprocessing was performed in MATLAB 2023a (MathWorks, Natick, MA, USA) via DPABI V9.0 (http://rfmri.org/DPABI) (22), which operates on the SPM12 platform. The preprocessing pipeline comprised: (1) removal of the first 10 time points, (2) slice timing correction, (3) three-dimensional rigid-body motion correction, and (4) spatial normalization to echo-planar imaging (EPI) template space with 3 × 3 × 3 mm³ resampling. Normalized images were smoothed with a 6-mm full-width-at-half-maximum (FWHM) Gaussian kernel followed by linear detrending. Nuisance covariates—including Friston-24 head motion parameters and white matter signals—were regressed from the fMRI time series. Temporal bandpass filtering (0.01–0.08 Hz) was subsequently applied. Volumetric outliers were scrubbed using framewise displacement (FD) thresholding (FD > 0.5 mm). Participants exhibiting excessive motion (>2.5 mm translation, >2.5° rotation, or >50% scrubbed volumes) were excluded from subsequent analyses. Functional networks were then constructed in DPABI using preprocessed fMRI data. The automated anatomical labeling (AAL) atlas parcellated the brain into 90 regions of interest (ROIs), serving as network nodes. For each subject, a 90 × 90 functional connectivity matrix was generated by computing Pearson correlation coefficients between regional time series.

Graph theoretical analysis was conducted using DPABINet 1.3 (http://rfmri.org/DPABI) to quantify topological properties of functional brain networks. Global topological metrics included: global efficiency (Eglob), local efficiency (Eloc), clustering coefficient (Cp), characteristic path length (Lp), normalized clustering coefficient (γ), normalized characteristic path length (λ), small-worldness scalar (σ), assortativity and modularity. Nodal topological properties (degree centrality, nodal efficiency, betweenness, and clustering coefficient) were additionally analyzed across all parcellated regions. We also computed the degree and link weight distribution for each patient group. The degree of a node was defined as the number of connections it possessed within the binary network. The degree distribution was extracted for each subject, and scatter plots were generated to compare the distributions between the two groups. The link weight distribution was defined as the probability distribution composed of the strength values of all existing functional connections. For each subject, all functional connectivity strength values were extracted from the functional connectivity matrix. Scatter plots were subsequently created to visualize and compare the link weight distributions of the two groups. Following established methodology (23), topological metrics were computed across a sparsity threshold range of 0.10–0.34 (incremental step=0.01) to ensure measurement robustness. For each topological metric, the area under the curve (AUC) was calculated over this sparsity range to generate threshold-insensitive integrated indices for network normalization.

### 2.5 Statistical analysis

Demographic and clinical characteristics between responders and non-responders were compared employing: Mann-Whitney U tests for non-normal continuous variables (age), chi-square tests for categorical variables (gender), and independent t-tests for normally distributed clinical scores (HAMD-17, HAMA). Group differences in network properties were assessed using nonparametric permutation tests on the AUC of each topological metric based on MATLAB. For each metric, all values were randomly assigned to two groups, and the inter-group mean difference was calculated. This randomization procedure was iterated 10,000 times, and the 95th percentile of each distribution was used as the critical value for a two-tailed test of the null hypothesis with a type I error of 0.05. Statistical significance for nodal measures was then corrected using false discovery rate (FDR) method (α=5%). We performed Kolmogorov-Smirnov (K-S) tests and Mann-Whitney U tests to statistically compare the degree and link weight distributions between the two groups. Partial correlation analyses-controlling for age and gender-examined relationships between network topology indices and symptom measures (Pre-treatment HAMD-17, ΔHAMD-17), with statistical significance defined as *P*<0.05. Significant global and nodal attributes identified through univariate regression subsequently underwent forward likelihood ratio (LR) binary logistic regression to identify SSRIs treatment response predictors.

## 3 Results

### 3.1 Demographic characteristics

Pre-treatment MRI and clinical data were collected from 69 adolescent MDD patients. Following 8 weeks of SSRIs treatment, 37 patients were classified as responders and 32 as non-responders. As presented in **Table 1**, the groups showed no statistically significant differences (*P* > 0.05) in age, sex distribution, Body Mass Index (BMI) or Pre-treatment scores on the HAMD-17 and HAMA scores.

**Table 1:** Demographic and clinical characteristics.

Characteristics	Responders(n=37)	Non-responders(n=32)	*P*-value
Age	15.57 ± 1.80	15.28 ± 1.20	0.383^1^
Sex(male/female)	14/23	6/26	0.081^2^
BMI	20.64 ± 3.14	21.43 ± 3.84	0.181^3^
Pre-treatment HAMD-17	18.32 ± 4.74	18.47 ± 6.26	0.914^3^
Pre-treatment HAMA	15.70 ± 6.67	15.03 ± 6.74	0.679^3^
After-treatment HAMD-17	3.68 ± 3.50	17.14 ± 6.60	<0.001
After-treatment HAMA	3.06 ± 3.20	14.69 ± 8.75	<0.001

HAMD-17, The 17-item Hamilton Depression Rating Scale; HAMA, Hamilton Anxiety Scale; ^1^Mann-Whitney U test; ^2^chi-square tests; ^3^t-tests.

### 3.2 Comparison of graph theory indicators

**Figure 1:**
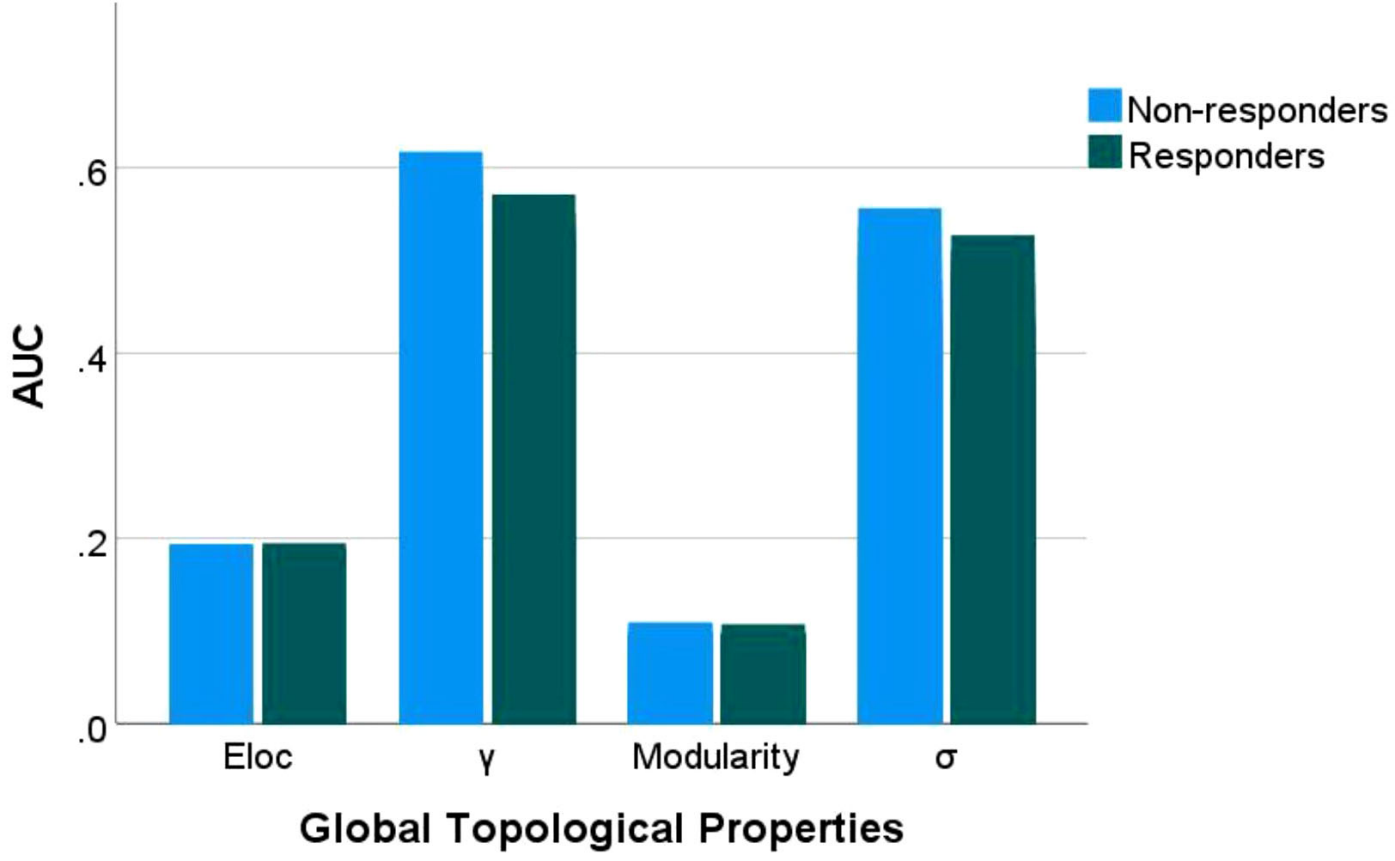
Group differences in global topological properties. Eloc: local efficiency; γ: normalized clustering coefficient; σ: small-worldness scalar. AUC, area under the receiver operating characteristic curve.

**Figure 2:**
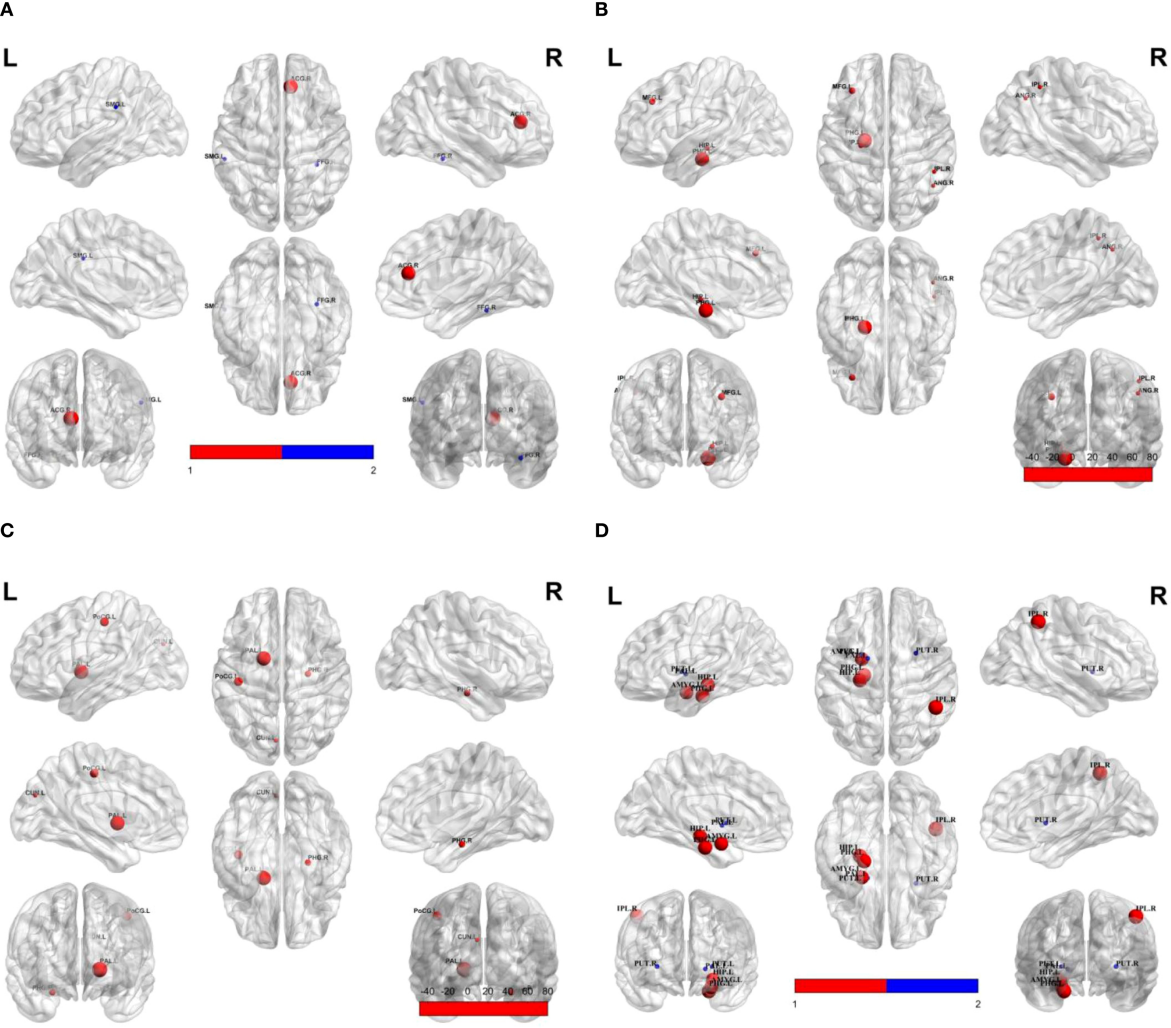
Group differences in nodal property metrics. Spheres represent brain regions showing significant differences between non-responders and responders. Red indicates higher values in non-responders; blue indicates lower values in non-responders relative to responders. **(A)** Degree centrality; **(B)** Nodal efficiency; **(C)** Betweenness; **(D)** Clustering coefficient. SMG.L, Left supramarginal gyrus; ACG.R, Right anterior cingulate gyrus; FFG.R, Right fusiform gyrus; MFG.L, Left middle frontal gyrus; HIP.L, Left hippocampus; PHG.L, Left parahippocampal gyrus; IPL.R, Right inferior parietal lobule; PoCG.L, Left postcentral gyrus; PAL.L, Left pallidum; CUN.L, Left cuneus; PHG.R, Right parahippocampal gyrus; PUT.L, Left putamen; AMYG.L, Left amygdala; PUT.R, Right putamen.

Compared to responders, non-responders exhibited significantly increased values in global network metrics including normalized Cp, Eloc, modularity, and σ (**Figure 1**, **Table 2**). At the nodal level: Higher nodal efficiency was observed in non-responders within the left middle frontal gyrus (MFG), left hippocampus, left parahippocampal gyrus, right inferior parietal lobule (IPL), and right angular gyrus. Elevated betweenness centrality occurred in the left pallidum, left postcentral gyrus, left cuneus, and right parahippocampal gyrus. Increased clustering coefficients were found in the left hippocampus, left parahippocampal gyrus, right IPL, and left amygdala but decreased in the bilateral putamen and left pallidum. Higher degree centrality was identified in the right anterior cingulate cortex (ACC), whereas reduced degree centrality was observed in both the right fusiform gyrus and the left supramarginal gyrus (*P <*0.05) (**Figure 2**, **Table 3**). For degree and link weight distribution, there was no statistically significant difference in degree distribution between the two patient groups, while the response group exhibited a higher link weight distribution. (see **Supplementary Figures 1**, **2**).

**Table 2:** Group differences in global topological properties.

Global topological properties	Responders	Non-responders	*P*-value
Clustering coefficient	0.1418 ± 0.0065	0.1445 ± 0.0048	0.058
Characteristic path length	0.4632 ± 0.0241	0.4606 ± 0.0157	0.613
Normalized Clustering coefficient	0.4463 ± 0.0640	0.4804 ± 0.0672	0.035^1^
normalized Characteristic path length	0.2644 ± 0.0079	0.2638 ± 0.0061	0.767
Small-worldness scalar	0.4029 ± 0.0634	0.4332 ± 0.0617	0.049^1^
Local efficiency	0.1808 ± 0.0055	0.1841 ± 0.0047	0.010^1^
Global efficiency	0.1277 ± 0.0058	0.1283 ± 0.0037	0.597
Assortativity	0.0482 ± 0.0173	0.0546 ± 0.0241	0.209
Modularity	0.0830 ± 0.0108	0.0889 ± 0.0108	0.029^1^

^1^ *P*<0.05.

**Table 3:** Group differences in nodal property metrics.

Nodal property metrics	Brain regions	*T*-value	*P*-value
Degree centrality	Right anterior cingulate cortex	2.40	0.019
	Right fusiform gyrus	-2.26	0.027
	Left supramarginal gyrus	-2.38	0.020
Nodal efficiency	Left middle frontal gyrus	2.23	0.029
	Left hippocampus	2.13	0.037
	Left parahippocampal gyrus	2.74	0.008
	Right inferior parietal lobule	2.07	0.042
	Right angular gyrus	2.07	0.042
Betweenness	Right parahippocampal gyrus	2.11	0.039
	Left cuneus	2.03	0.046
	Left postcentral gyrus	2.19	0.032
	Left pallidum	2.38	0.020
Clustering coefficient	Left hippocampus	2.40	0.019
	Left parahippocampal gyrus	2.41	0.019
	Left amygdala	2.17	0.034
	Right inferior parietal lobule	2.61	0.011
	Left putamen	-2.45	0.017
	Right putamen	-2.17	0.034
	Left pallidum	-2.21	0.031

### 3.3 Correlation with clinical symptoms

**Figure 3:**
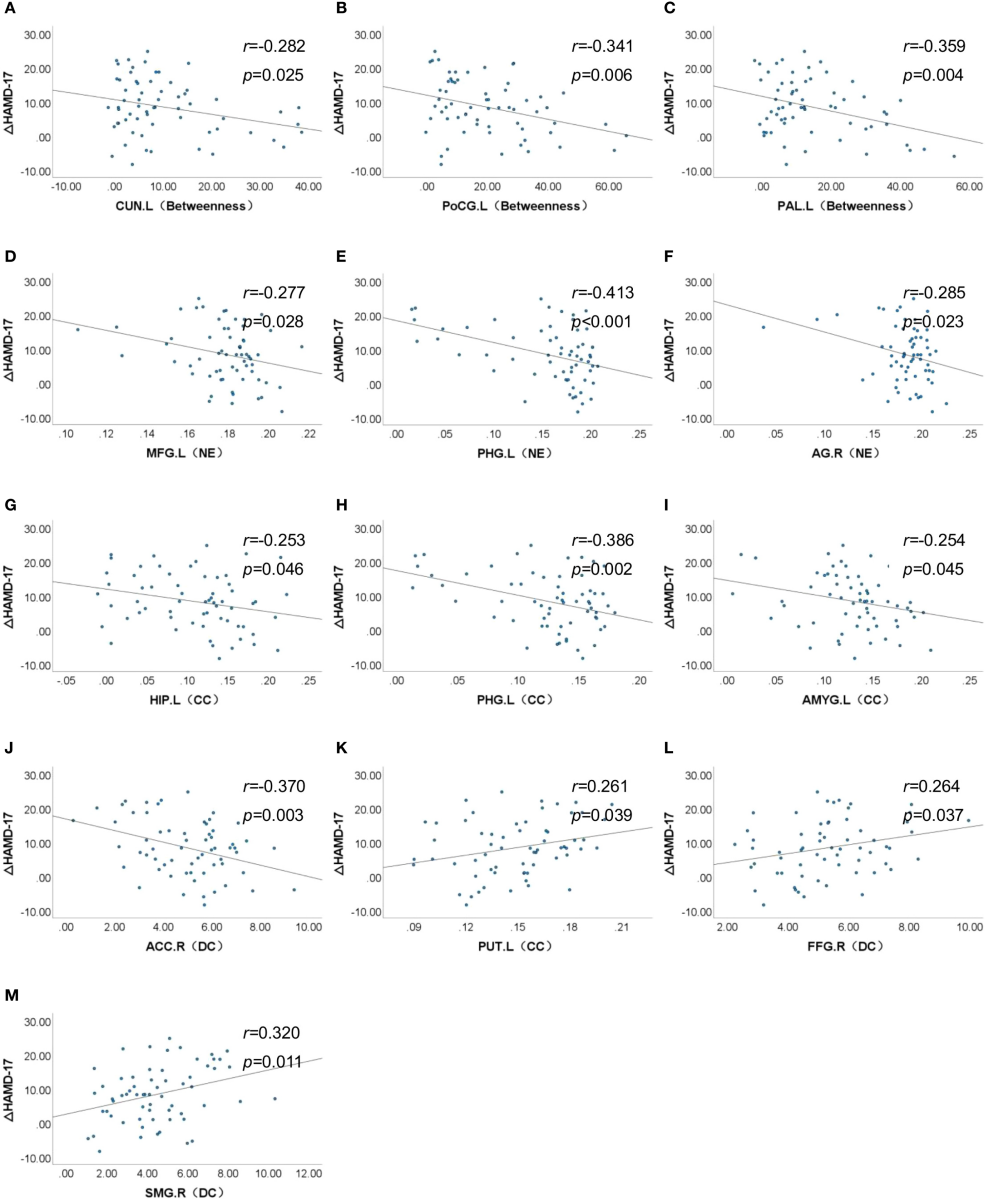
Association between nodal properties and ΔHAMD-17 scores. ΔHAMD-17: Pre-treatment HAMD-17 score minus Post-treatment HAMD-17 score; **(A)** CUN.L (Betweeness), Betweeness in the left cuneus; **(B)** PoCG.L (Betweeness), Betweeness in the left postcentral gyrus; **(C)** PAL.L (Betweeness), Betweeness in the left postcentral gyrus; **(D)** MFG.L (NE), Nodal efficiency in the left middle frontal gyrus; **(E)** PHG.L (NE), Nodal efficiency in the left parahippocampal gyrus; **(F)** AG.R (NE), Nodal efficiency in the right angular gyrus; **(G)** HIP.L (CC), Clustering coefficient in the left hippocampus; **(H)** PHG.L (CC), Clustering coefficient in the left parahippocampal gyrus; **(I)** AMYG.L (CC), Clustering coefficient in the left amygdala; **(J)** ACC.R (DC), Degree centrality in the right anterior cingulate cortex; **(K)** PUT.L (CC), Clustering coefficient in the left putamen; **(L)** FFG.R (DC), Degree centrality in the right fusiform gyrus; **(M)** SMG.R (DC), Degree centrality in the left supramarginal gyrus.

**Figure 4:**
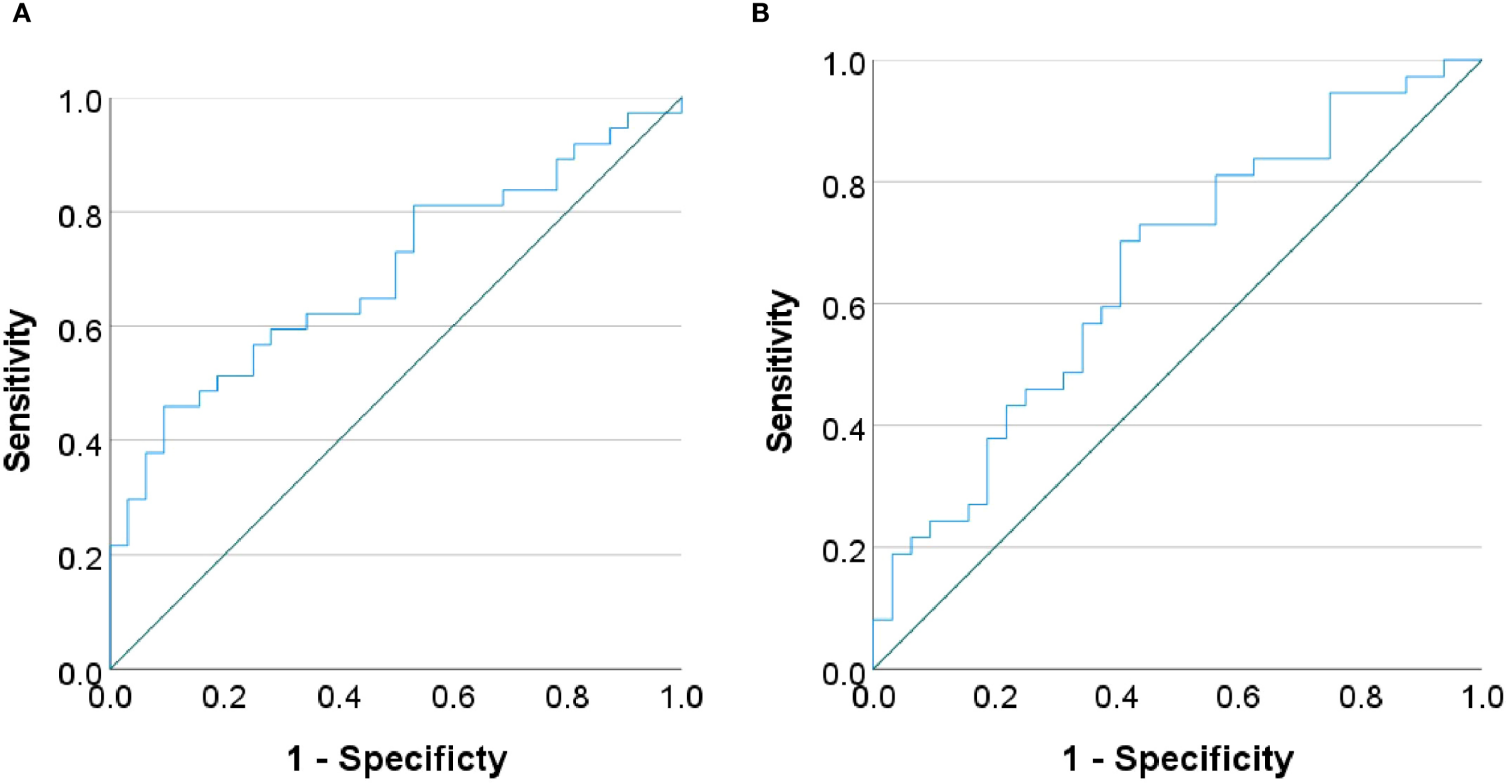
Receiver operating characteristic (ROC) curves. The curves demonstrating the performance of **(A)** nodal efficiency in the right inferior parietal lobule and **(B)** clustering coefficient in the left pallidum in predicting treatment response.

Partial correlation analyses controlling for age and sex revealed no significant associations between global network metrics and ΔHAMD-17 scores (*P* > 0.05). However, nodal metrics demonstrated significant correlations: betweenness centrality in the left cuneus, left postcentral gyrus and left pallidum; nodal efficiency in the left MFG, left hippocampus, left parahippocampal gyrus and right angular gyrus; and degree centrality in the right ACC showed negative correlations with ΔHAMD-17 (all *P*<0.05). Conversely, positive correlations were observed for the clustering coefficient in the left putamen and degree centrality in the right fusiform gyrus and left supramarginal gyrus (all *P*<0.05). Critically, nodal efficiency in the left MFG and degree centrality in the right ACC were negatively correlated with absolute HAMD-17 scores (*P*<0.05)(**Figure 3**). Subsequent binary logistic regression identified lower nodal efficiency in the right IPL (sensitivity=0.459, specificity=0.906, AUC=0.692) and higher clustering coefficient in the left pallidum (sensitivity=0.703, specificity=0.594, AUC=0.655) as significant predictors of 8-week SSRIs treatment response (**Figure 4**, **Table 4**).

**Table 4:** Binary logistic regression analysis of nodal property metrics.

Nodal property metric	OR(95%CI)	*P*-value
Right inferior parietal lobule nodal efficiency	0.002(0.000~0.258)	0.012
Left pallidum clustering coefficient	54.944(1.626~1857.049)	0.026

OR (95%CI): Odds ratios (OR) and 95% confidence intervals (95% CI) for predicting treatment response compared to the non-responders group.

## 4 Discussion

This resting-state fMRI study identified potential biomarkers predictive of SSRIs treatment response by comparing Pre-treatment functional brain network topology between treatment responders and non-responders in first-episode, drug-naïve adolescents with MDD. We detected significant between-group differences in global network properties and nodal metrics across key brain regions at pre-treatment. Critically, lower nodal efficiency in the right IPL and higher clustering coefficients in the left pallidum emerged as predictors of treatment response. These findings demonstrate that graph-theoretic analysis effectively captures treatment-predictive topological features in brain networks.

This study reveals that non-responders to SSRIs exhibit treatment significantly elevated global network segregation metrics (γ, Eloc, modularity) compared to responders. Furthermore, non-responders showed a pronounced rise in σ and a notable reduction in link weight distribution. This decrease in link weight distribution may reflect a relative loss of redundant connections, which could potentially improve the efficiency of information integration within brain networks. This finding aligns with enhanced small-world characteristics in the non-responders. These results point to a more severe segregation-integration imbalance potentially underlying pharmacoresistance in non-responders (24, 25). Supporting evidence links global topology to SSRIs efficacy: Wang et al. demonstrated negative correlations between pre-treatment Lp, λ and HAMD-17 reduction after 8-week antidepressant treatment in adult MDD, while Eglob showed a positive correlation (26). Similarly, Nakamura et al. observed increased small-world efficiency and modularity paralleling clinical improvement in escitalopram-treated obsessive-compulsive disorder (OCD) patients after 16 weeks (27). Collectively, these results-including ours-position global topological metrics as transdiagnostic predictors of SSRIs response. Nevertheless, significant heterogeneity across studies necessitates large-sample longitudinal validation through graph-theoretic approaches.

Our findings revealed that elevated nodal efficiency in the left MFG of non-responders, indicating enhanced local information integration, is associated with unfavorable treatment outcomes. The MFG, a core component of the dorsolateral prefrontal cortex (DLPFC), plays well-established roles in cognitive control, working memory, and emotion regulation (28–30). Convergent longitudinal evidence demonstrates that escitalopram responders exhibit reduced resting-state functional connectivity (rsFC) between the right DLPFC and left MFG after 8-week treatment, while non-responders show no such change (10). Similarly, reduced left MFG activation during verbal working memory (VWM) task-fMRI at baseline correlates with better clinical outcomes (31), and structural MRI studies associate greater left MFG grey matter density with favorable SSRIs response (32). Additionally, we observed between-group differences in the betweenness centrality of the left postcentral gyrus, nodal efficiency and clustering coefficient of the right IPL- regions comprising the frontoparietal control network (FPCN) with the left MFG. The FPCN mediates executive control and coordinates goal-directed behaviors (33, 34). Notably, pre-treatment thalamo-FPCN functional connectivity predicts sertraline outcomes in adult MDD with moderate accuracy (R²=0.63) (35), further supporting FPCN’s predictive utility for SSRIs response. Collectively, these findings highlight both the left MFG and its embedded FPCN circuitry as critical neural substrates influencing SSRI response, warranting future multicenter studies that integrate these multimodal features via machine learning into robust, precision treatment prediction models.

Our study reveals elevated degree centrality in the right anterior cingulate cortex (ACC) of SSRI non-responders compared to responders, indicating hyperintegration of information processing in this key region for emotional regulation. The ACC mediates emotional regulation through its coordination of cognitive and affective processes within the prefrontal circuitry (36, 37). What we found aligns with task-based fMRI evidence linking ACC overactivation to poor antidepressant outcomes: symptom improvement in SSRI-treated adolescent MDD correlates with reduced activation in the rostral subgenual ACC during negative emotion tasks (38). Convergent evidence demonstrates that lower pre-treatment ACC responses during verbal working memory (VWM) tasks predict better clinical outcomes (31), while heightened pre-treatment ACC-amygdala connectivity during negative emotional processing predicts treatment non-response (39). Critically, ACC metrics show direct predictive utility: pre-treatment subgenual ACC (sgACC) resting-state functional connectivity achieves 72.64% accuracy in predicting 12-week escitalopram response (40), and support vector machine (SVM) models incorporating ACC features achieved 79.41% accuracy for SSRIs response prediction (41). Collectively, these findings establish pre-treatment ACC functional features—particularly its hyperconnectivity—as a robust predictor of SSRI treatment response, offering significant potential for guiding personalized treatment selection.

Our findings reveal elevated clustering coefficients in the left amygdala of SSRI non-responders compared to responders, indicating altered local functional integration within this key affective hub, which aligns with convergent evidence linking aberrant left amygdala activity and connectivity to poor antidepressant outcomes. The amygdala serves as a hub of the affective network (AN), critically enabling higher-order emotional processing including emotion perception, emotional memory formation, and affect regulation (42, 43). Evidence indicates left amygdala dominance, with preferential activation during affective challenges and unique responsiveness to top-down regulation (44, 45), suggesting heightened susceptibility to prefrontal modulation in affective pathology. Our finding aligns with reports that reduced left amygdala activation during masked emotional face tasks predicts superior citalopram response at 8 weeks (46), while pre-treatment hyperconnectivity between the amygdala and left supplementary motor area (SMA) correlates with SSRI non-response in adolescent MDD (47). Collectively, these differential left amygdala alterations hold promise for predicting SSRI efficacy. Future multicenter longitudinal studies should leverage multimodal MRI (structural, functional, connectomic) and machine learning to validate amygdala-based biomarkers for SSRI response prediction.

This study identifies elevated nodal efficiency and clustering coefficient in the left hippocampus/parahippocampal gyrus of SSRI non-responders compared to responders, suggesting aberrant local functional integration and heightened network engagement within these core limbic structures, which aligns with convergent evidence implicating left hippocampal/parahippocampal alterations in poor treatment outcomes. The hippocampus and parahippocampal gyrus constitute core limbic structures essential for emotion regulation, memory encoding and retrieval (36, 38, 48). We observed elevated nodal efficiency and clustering coefficient in the left hippocampus/parahippocampal gyrus of non-responders, suggesting aberrant local functional integration and heightened network engagement. This aligns with task-fMRI evidence linking reduced left hippocampal activation during positive word-pair encoding to poor treatment response (49). Structural MRI studies further associate baseline gray matter density/volume in these regions with SSRI outcomes (32, 50). However, divergent findings exist-such as positive correlations between left hippocampal nodal efficiency and early antidepressant symptom changes in adult MDD (51) - potentially attributable to treatment duration, illness chronicity, or sample size limitations. Additionally, non-responders exhibited increased nodal efficiency in the right angular gyrus and decreased degree centrality in the left supramarginal gyrus-regions belonging to the default mode network (DMN) alongside the hippocampal complex. The DMN supports self-referential processing, autobiographical memory, and social cognition (52, 53), with its hyperactivity potentially reflecting pathological self-focus in MDD. Crucially, DMN connectivity patterns (intra- and inter-network) demonstrate predictive utility for SSRIs treatment efficacy (54–57), solidifying its role as a key predictor. Collectively, these findings highlight both the left hippocampal/parahippocampal and their embedded DMN circuitry as potential SSRI response predictors. Future large-scale longitudinal studies integrating dynamic network analysis and machine learning should establish robust biomarkers.

Critically, this study identifies distinct pre-treatment alterations in the right inferior parietal lobule (IPL) and left pallidum as potential predictors of SSRI treatment response, with lower nodal efficiency in the right IPL and higher clustering coefficient in the left pallidum specifically associated with favorable outcomes. Non-responders exhibited elevated nodal efficiency in the right IPL but reduced clustering coefficient in the left pallidum compared to responders. The IPL mediates fundamental attention, language, and social cognition (58). Relevant evidence demonstrates IPL’s predictive relevance: structural MRI reveals significantly greater post-treatment cortical thickness increases in the right IPL of remitters after 8-week antidepressant therapy (59), while task-fMRI shows greater pre-treatment right IPL activation during Go/No Go tasks in eventual SSRIs remitters (60). Similarly, the pallidum contributes to emotional processing and antidepressant neuromodulation (61). Strikingly analogous to our results, fluoxetine responders exhibit a transient metabolic increase followed by a decrease in the left pallidum (62), with pre-treatment temporal variability in this region predicting 2-week HAMD reduction (r=0.62, *P*<0.01) (63). These converging lines of evidence establish both right IPL and left pallidum as robust predictors of SSRIs efficacy.

This study has several limitations. First, although all participants received SSRIs as primary treatment, potential confounding effects from concomitant medications (e.g.mirtazapine, buspirone) cannot be fully excluded in real-world clinical settings. Future studies should implement stricter enrollment criteria, medication stratification, or subgroup analyses to address this limitation. Second, the moderate sample size (n=69) constrains generalizability; multicenter collaborations with expanded cohorts are essential. Third, it should be noted that spatial smoothing was applied during preprocessing. This process may influence the estimation of the functional connectivity matrix and consequently bias the subsequent calculation of network metrics (64). To further demonstrate the robustness of our results, we repeated the analyses using unsmoothed data. The statistical results were similar to those obtained with smoothed data, suggesting that smoothing did not unduly affect our conclusions (see **Supplementary Table 1**). Fourth, the use of the AAL atlas for parcellation represents another potential limitation, as the choice of atlas can influence estimates of network topology (65). Future studies should therefore compare graph-theoretic results across multiple parcellation schemes. Fifth, the exclusive reliance on baseline MRI data precludes longitudinal assessment; future work should incorporate serial neuroimaging to establish causal relationships between network dynamics and treatment outcomes.

## 5 Conclusion

This study pioneers graph-theoretical analysis of pre-treatment functional brain networks to predict SSRI response in first-episode, drug-naïve adolescent MDD, revealing significant topological differences between responders and non-responders. Critically, reduced nodal efficiency in the right IPL and elevated clustering coefficient in the left pallidum emerged as key predictors of treatment response. These topology-based features offer potential neuroimaging indicators for precision medicine. Future multicenter longitudinal studies should validate these predictors using multimodal MRI, while machine learning frameworks integrating neuroimaging, genomics, and metabolomics could elucidate biological mechanisms and advance precision psychiatry for MDD.

## Data Availability

The raw data supporting the conclusions of this article will be made available by the authors, without undue reservation.
