# Supplementary figures and images for: Resting-state fMRI graph theory analysis for predicting selective serotonin reuptake inhibitors treatment response in adolescent major depressive disorder
Source: Front Psychiatry. 2025 Oct 1;16:1675719. doi: 10.3389/fpsyt.2025.1675719 (PMC12521158; doi:10.3389/fpsyt.2025.1675719)

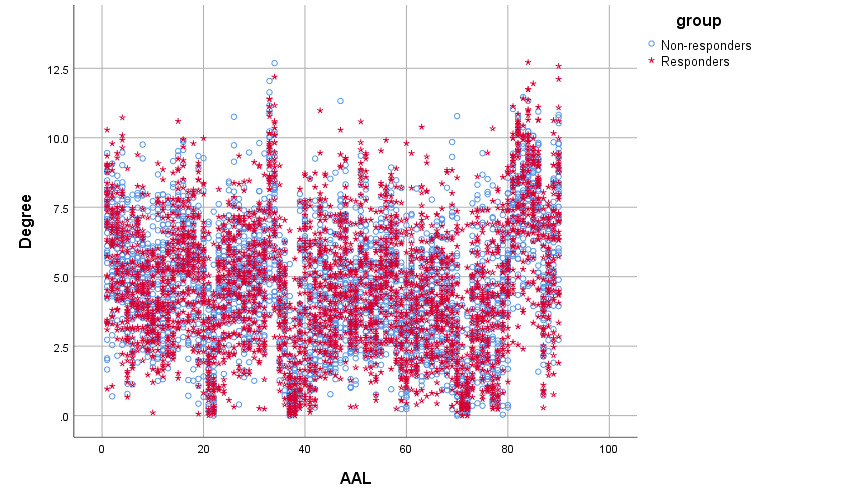

Supplement: Supplementary file 1 [file Image1.jpeg]

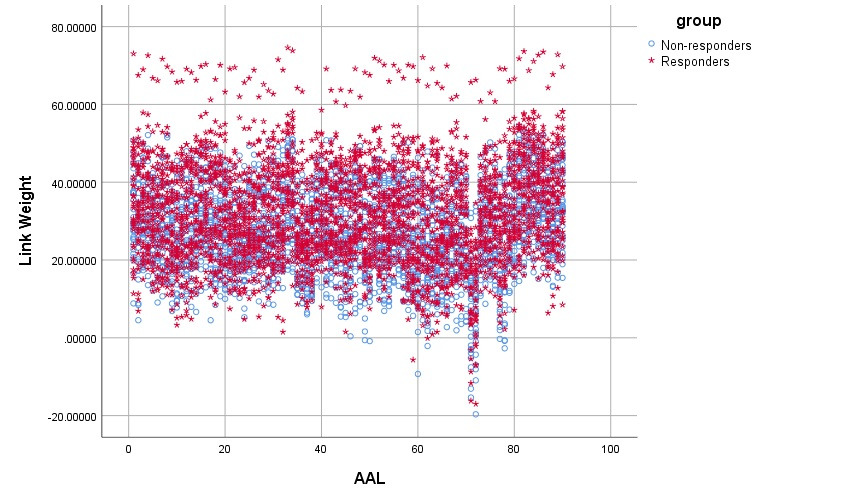

Supplement: Supplementary file 2 [file Image2.jpeg]

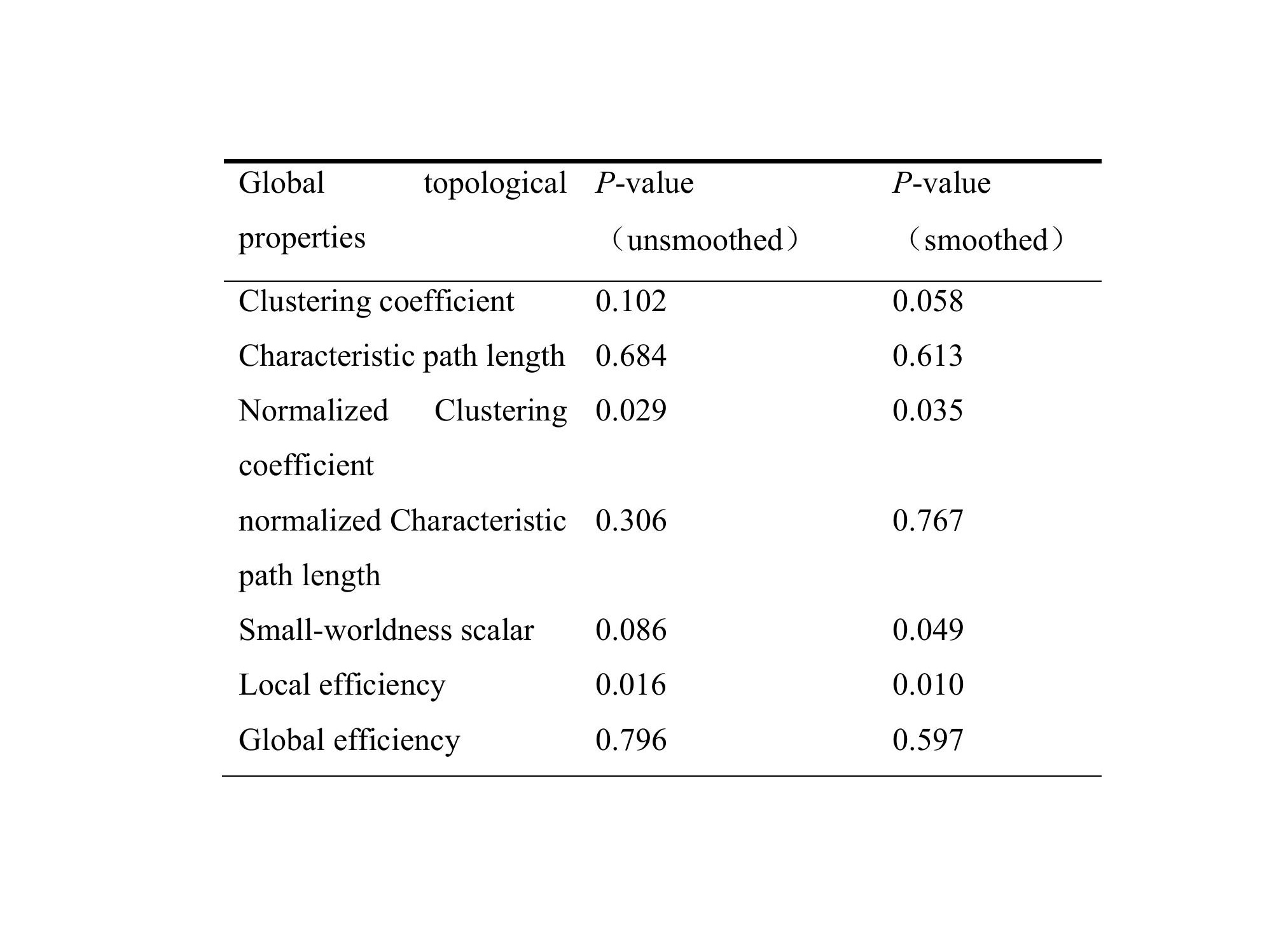

Supplement: Supplementary file 3 [file Image3.jpeg]
